# Ectopic Thyroid Tissue Mimicking a Thyroglossal Duct Cyst in a Child: A Diagnostic Pitfall Leading to Postoperative Hypothyroidism

**DOI:** 10.7759/cureus.116705

**Published:** 2026-09-22

**Authors:** Ayush Tank, Khushboo G Malhotra, Jahanvi Desai, Ajeet K Khilnani, Narendra Hirani

**Affiliations:** 1 Otolaryngology-Head and Neck Surgery, Gujarat Adani Institute of Medical Sciences, Bhuj, IND

**Keywords:** ectopic thyroid tissue, pediatric neck mass, sistrunk’s procedure, sub-clinical hypothyroidism, thyroglossal duct cyst mimic, thyroid scintigraphy

## Abstract

Ectopic thyroid tissue is an uncommon developmental anomaly resulting from abnormal migration of the thyroid gland. It may occur anywhere along the embryological descent pathway and can clinically mimic a thyroglossal duct cyst (TGDC). Recognition before surgery is particularly important because ectopic thyroid tissue may represent the patient's only functioning thyroid tissue, and its inadvertent excision can result in hypothyroidism. A 10-year-old girl presented with a painless midline neck swelling that had been present since childhood and had increased slightly in size over the preceding year. The swelling moved with deglutition and tongue protrusion, and a clinical diagnosis of TGDC was made. Preoperative thyroid function testing showed mildly elevated thyroid-stimulating hormone (TSH) of 5.455 µIU/mL (normal range 0.4 to 4.0 µIU/mL). Fine-needle aspiration cytology was reported as Bethesda category II, consistent with a benign cystic thyroid nodule. Ultrasonography suggested a thyroid lesion, while contrast-enhanced computed tomography demonstrated a 29 × 32 × 36 mm solid-cystic lesion anterior and inferior to the hyoid bone, with the possibility of a TGDC containing ectopic thyroid tissue. Importantly, the normally located thyroid gland was not visualized. A separate 8 × 7 mm hyperdense lesion at the tongue base was considered radiologically suggestive of ectopic thyroid tissue. The patient underwent Sistrunk's procedure. Histopathological examination unexpectedly confirmed ectopic thyroid tissue. On postoperative day seven, TSH increased markedly to 99.65 µIU/mL, and levothyroxine therapy was initiated. Subsequent Tc-99m pertechnetate thyroid scintigraphy demonstrated no tracer uptake in the thyroid bed or at recognized ectopic sites. Ectopic thyroid tissue should be considered in the differential diagnosis of a midline neck swelling clinically suggestive of TGDC, particularly when the normally located thyroid gland is not visualized. Identification of the anatomical and functional status of thyroid tissue before surgery is essential to minimize the risk of postoperative hypothyroidism. This case emphasizes the importance of integrating clinical, radiological, cytological, and functional assessment before excision of presumed TGDCs in children.

## Introduction

The thyroid gland originates from an endodermal thickening at the foramen cecum at the base of the tongue and subsequently descends caudally along the midline through the thyroglossal duct to reach its definitive pre-tracheal position by approximately the seventh week of gestation [1]. Abnormalities in this developmental migration may result in ectopic thyroid tissue, a rare congenital anomaly with an estimated prevalence of approximately one in 100,000-300,000 individuals [2]. Although the tongue base is the most common site of ectopic thyroid tissue, ectopic thyroid may occur at any point along the normal pathway of thyroid descent, including submandibular, subhyoid, and mediastinal locations [2].

Ectopic thyroid tissue may remain asymptomatic or present with dysphagia, foreign-body sensation, airway symptoms, or enlargement of the ectopic tissue [3,4]. An important clinical consideration is that ectopic thyroid tissue may constitute the only functioning thyroid tissue in a substantial proportion of affected individuals [3]. Consequently, inadvertent surgical excision of ectopic thyroid tissue may result in permanent hypothyroidism and the need for lifelong thyroid hormone replacement.

Thyroglossal duct cysts (TGDCs) arise from persistent remnants of the thyroglossal duct and are among the most common congenital neck lesions. Ectopic thyroid tissue can occur in the anterior neck and mimic a TGDC. They typically present as painless midline neck swellings that move with swallowing and tongue protrusion [4,5]. Sistrunk's procedure is the surgical treatment of choice for TGDCs [6]. Because ectopic thyroid tissue may occur along the same embryological pathway, it can closely mimic a TGDC clinically and radiologically. Ectopic thyroid tissue has been reported in a small proportion of lesions clinically diagnosed as TGDCs [7]. Excision of unsuspected ectopic thyroid tissue can therefore have significant consequences, particularly in children, in whom prolonged thyroid hormone deficiency may adversely affect growth and neurodevelopment [7,8].

Preoperative assessment using ultrasonography, computed tomography (CT), magnetic resonance imaging, thyroid scintigraphy, and, in selected cases, fine-needle aspiration cytology (FNAC) can help differentiate ectopic thyroid tissue from other congenital neck lesions [9]. However, imaging modalities provide complementary anatomical information, whereas radionuclide scintigraphy provides functional information about thyroid tissue.

We report a case of a 10-year-old girl with a midline neck swelling clinically diagnosed as a TGDC, in whom imaging demonstrated ectopic thyroid tissue and the absence of the normally located thyroid gland. The patient underwent Sistrunk's procedure, and histopathological examination revealed ectopic thyroid tissue. She subsequently developed marked hypothyroidism requiring levothyroxine therapy. This case highlights the importance of establishing the anatomical and functional status of thyroid tissue before surgical excision of a presumed TGDC.

## Case presentation

A 10-year-old girl presented to the Department of Otorhinolaryngology at GK General Hospital, Bhuj, Gujarat, with a painless midline neck swelling that had been present since childhood. The swelling moved with deglutition and tongue protrusion and had increased slightly in size over the preceding 12 months. There was no history of dysphagia, odynophagia, dyspnea, or hoarseness. She did not report symptoms suggestive of thyroid dysfunction, including fatigue, weight gain, or cold intolerance. Her medical history was unremarkable, with normal developmental milestones and no family history of thyroid disorders. There was no history of radiation exposure or use of goitrogens. No clinically significant cervical or peripheral lymphadenopathy was noted. Based on the clinical presentation, a provisional diagnosis of thyroglossal duct cyst (TGDC) was made.

Ultrasonography of the neck demonstrated a well-defined, wider-than-tall, smooth-marginated, spongiform, isoechoic lesion without calcification involving the right lobe of the thyroid, which was reported as Thyroid Imaging Reporting and Data System (TIRADS I) [10]. Preoperative thyroid function testing showed a mildly elevated thyroid-stimulating hormone (TSH) level of 5.455 µIU/mL (normal range 0.4 to 4.0 µIU/mL), consistent with subclinical hypothyroidism. Fine-needle aspiration cytology (FNAC) was reported as Bethesda category II (benign), consistent with a cystic thyroid nodule [11].

Multi-slice computed tomography (MSCT) of the neck demonstrated a well-circumscribed, oval-shaped solid-cystic lesion measuring 29 × 32 × 36 mm (anteroposterior × transverse × craniocaudal) in the upper neck, anterior and inferior to the hyoid bone. The solid component was homogeneous and mildly enhancing following contrast administration and measured approximately 24 × 15 mm in the axial plane. The cystic component demonstrated enhancing internal septations, with a few peripheral foci of calcification. The lesion extended from the upper border of the C3 vertebra to the lower border of the C6 vertebra (Figure 1).

**Figure 1:**
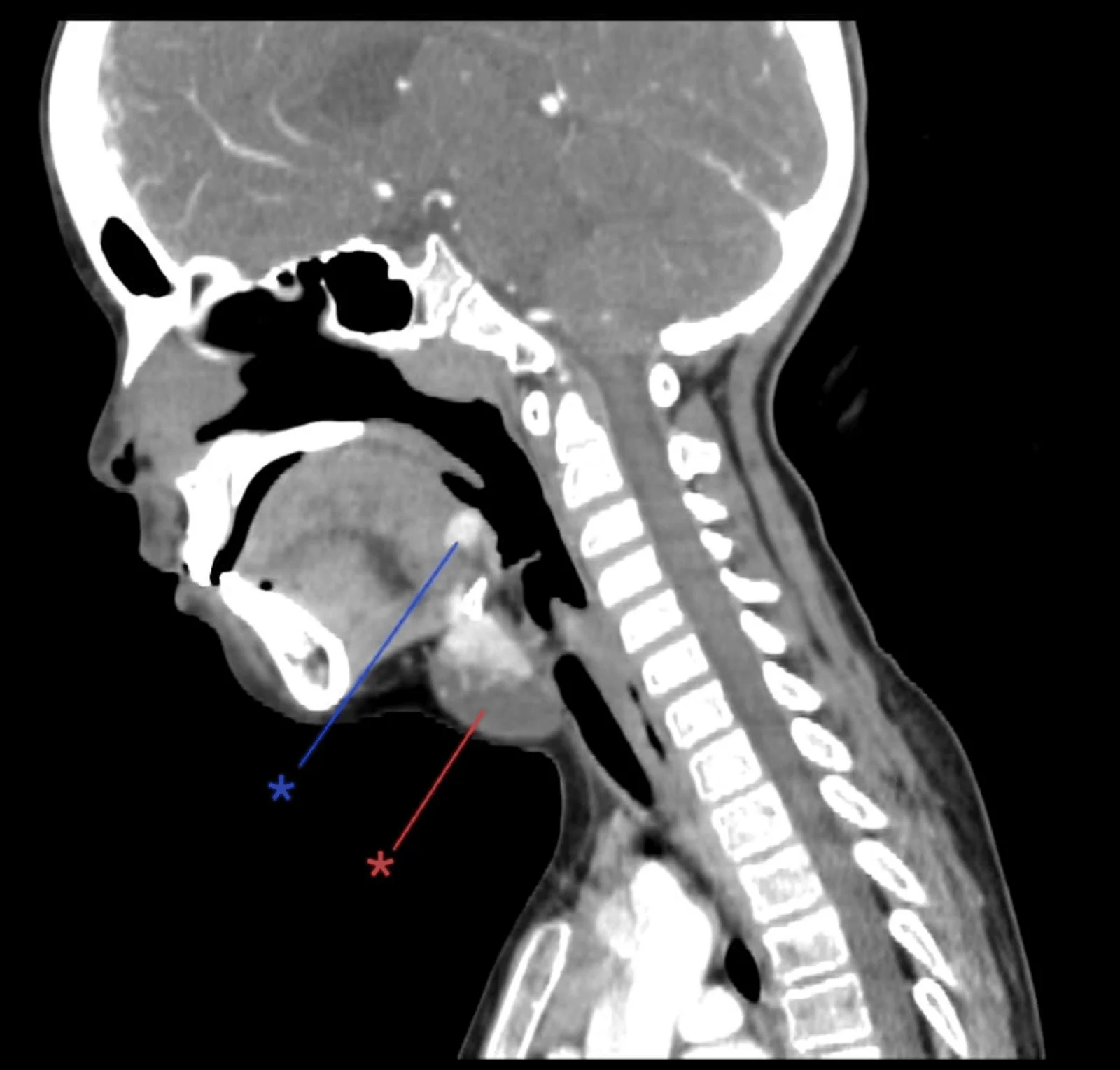
CECT showing well-circumscribed oval-shaped solid-cystic lesion of 29 x 32 x 36 mm (shown in red star) in size projecting from upper neck region anterior and inferior to hyoid bone and, a hypodense lesion seen at base of tongue (shown in blue star), possibility of ectopic thyroid gland. CECT: contrast-enhanced computed tomography.

The radiological impression favored a thyroglossal duct cyst with ectopic thyroid tissue. Importantly, the thyroid gland was not visualized in its normal anatomical location. A separate hyperdense lesion measuring 8 × 7 mm was identified at the base of the tongue and was considered likely to represent ectopic thyroid tissue. No communication was identified between the upper-neck lesion and the tongue-base lesion. There was no significant cervical lymphadenopathy.

In view of the clinical and radiological findings, the patient underwent Sistrunk's procedure under general anesthesia. A horizontal incision was made over the anterior neck corresponding to the palpable cystic lesion. The skin and subcutaneous tissues were dissected, followed by elevation of subplatysmal flaps. The strap muscles were separated, and the cyst tract was identified and dissected from the surrounding tissues. The central portion of the hyoid bone was excised along with the lesion, and the specimen was sent for histopathological examination. Hemostasis was achieved, a No. 12 drain was placed, and the wound was closed in layers. There were no immediate postoperative complications.

Histopathological examination revealed well-encapsulated thyroid parenchyma composed of thyroid follicles of variable size and shape arranged in microfollicular and macrofollicular patterns. Benign follicular cells with bland nuclei lined the follicles, which contained abundant colloid. Numerous colloidophages were present. Sections also demonstrated hyoid bone. The processed tissue showed no evidence of a TGDC remnant or cyst lining. The overall histopathological findings were consistent with ectopic thyroid tissue (Figure 2).

**Figure 2:**
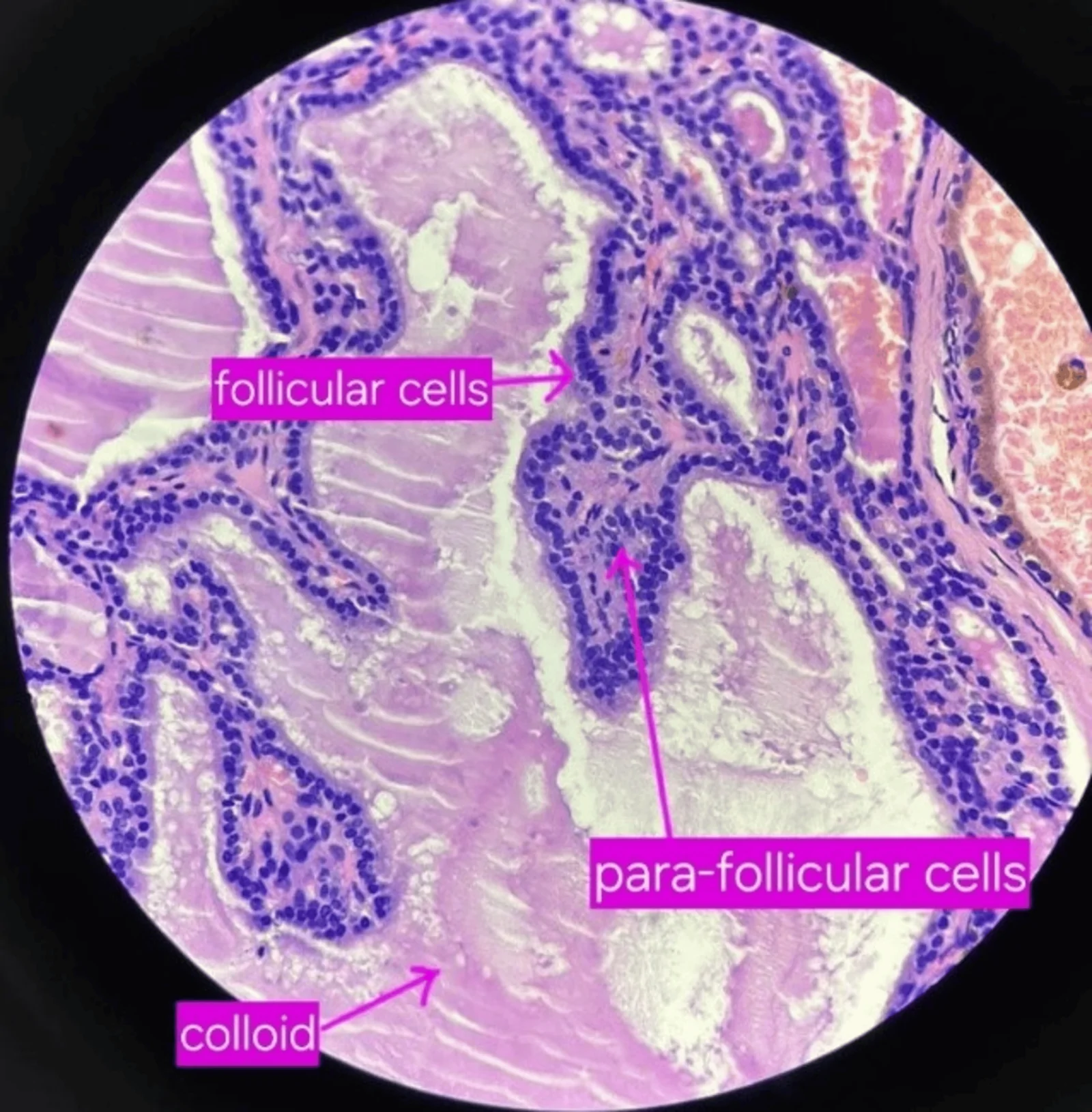
Histopathological (H&E, X100) suggestive of well-encapsulated thyroid parenchyma having thyroid follicles.

On the seventh postoperative day, thyroid function testing showed marked hypothyroidism, with a TSH of 99.65 µIU/ml, serum T3 of 0.662 ng/ml (normal range 0.820 to 1.58 ng/ml), and T4 of 2.78 µg/dl (normal range 5.92 to 12.97 µg/dl). The patient was started on levothyroxine 100 µg daily and is currently under three-monthly follow-up.

Two months after surgery, a Tc-99m pertechnetate thyroid scintigraphy was performed to evaluate the presence and functional status of thyroid tissue. Levothyroxine was stopped one week prior to thyroid scintigraphy. No tracer uptake was demonstrated in the region of the thyroid gland or elsewhere at recognized ectopic sites, including the clinically palpable neck swelling (Figure 3). Thus, no functioning thyroid tissue was demonstrated scintigraphically. 

**Figure 3:**
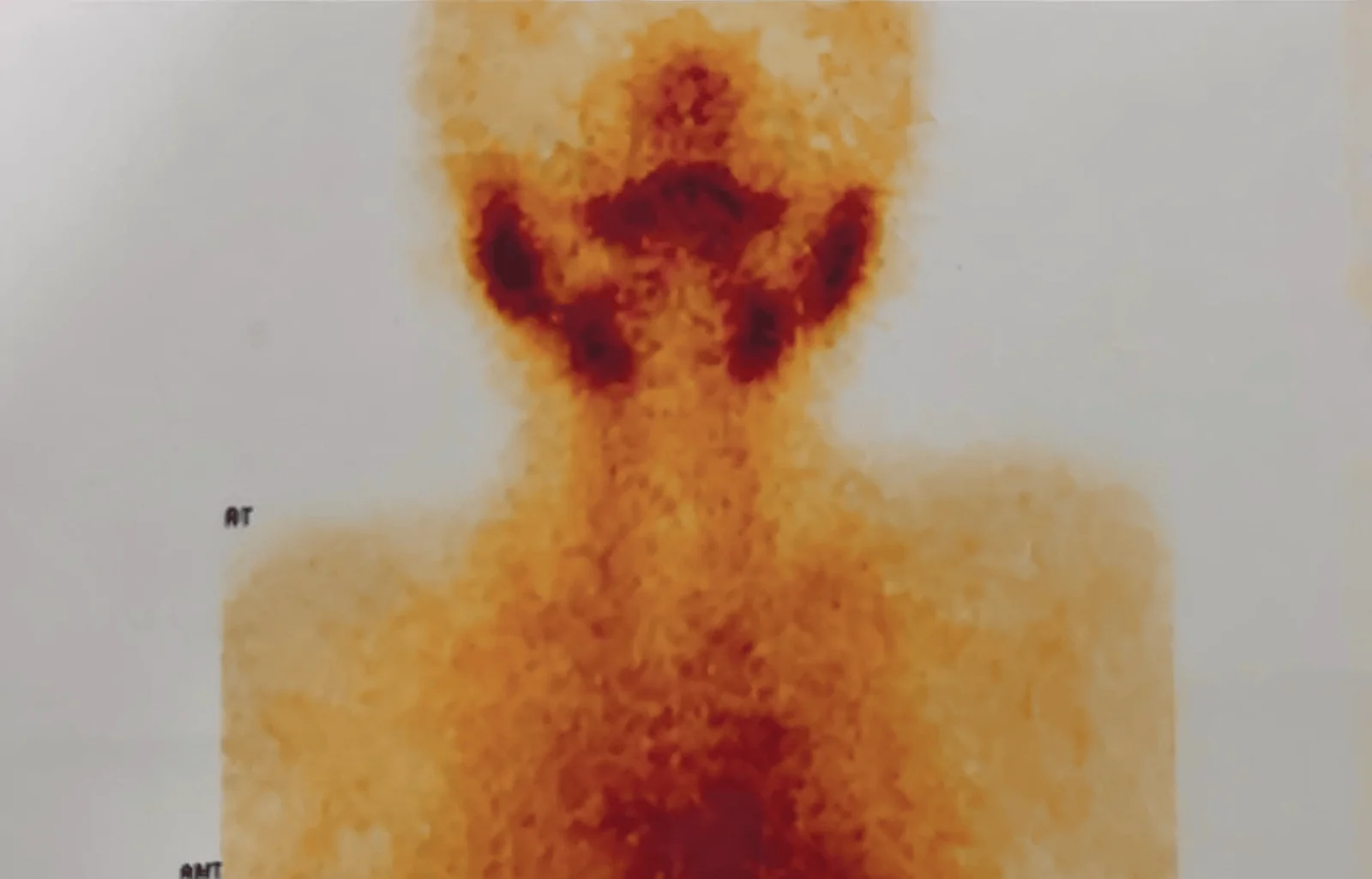
Postoperative Tc99m-pertechnetate thyroid scan suggestive of no functioning thyroid tissue in the scanned area.

## Discussion

Ectopic thyroid tissue masquerading as a thyroglossal duct cyst represents an important diagnostic pitfall because both conditions may present as painless midline neck swellings that move with deglutition and tongue protrusion [9,12,13]. The clinical presentation in our patient was characteristic of a TGDC, and the initial clinical diagnosis was therefore a TGDC.

The present case emphasizes the importance of determining both the anatomical location and functional status of thyroid tissue before excision of a presumed TGDC. Ultrasonography is generally the initial imaging modality for evaluating congenital midline neck masses and can identify thyroid tissue based on its characteristic echotexture, as described in the TI-RADS classification [10]. However, ectopic thyroid tissue may have variable sonographic appearances and may be difficult to distinguish from other neck lesions [9]. FNAC follows the Bethesda system and may demonstrate thyroid follicular cells and establish the thyroidal nature of a lesion, but cytology cannot determine whether the identified tissue represents the patient's only functioning thyroid tissue [11,14].

In the present case, the preoperative investigations provided several important clues. Although ultrasonography and FNAC initially characterized the lesion as a thyroid-associated benign cystic lesion, MSCT demonstrated that the major lesion was located in the upper neck, anterior and inferior to the hyoid bone. More importantly, the normally located thyroid gland was not visualized. A separate 8 × 7 mm hyperdense lesion at the base of the tongue was also considered radiologically suggestive of ectopic thyroid tissue. No communication between the two lesions was identified.

The absence of the thyroid gland at its expected anatomical location should be regarded as a significant warning sign before surgery. When ectopic thyroid tissue is suspected, radionuclide thyroid scintigraphy with Tc-99m pertechnetate can provide valuable functional information and help identify functioning thyroid tissue [15,16]. In contrast to CT or MRI, which primarily demonstrate anatomy, scintigraphy demonstrates thyroid tissue capable of tracer uptake and can therefore help determine whether a suspected ectopic focus is functioning.

In our patient, postoperative scintigraphy demonstrated no tracer uptake in the thyroid bed or at the recognized ectopic sites. Thus, the tongue-base lesion identified on CT could not be confirmed as functioning thyroid tissue. Nevertheless, histopathological examination of the excised neck lesion unequivocally demonstrated thyroid tissue. The functional importance of the excised tissue was further supported by the patient's biochemical course.

Preoperatively, the patient's TSH was mildly elevated at 5.455 µIU/mL, indicating subclinical hypothyroidism. Following excision of the ectopic thyroid tissue, the TSH increased markedly to 99.65 µIU/mL by the seventh postoperative day. The rapid development of marked hypothyroidism following excision strongly suggests that the removed ectopic thyroid tissue had been contributing substantially to the patient's thyroid hormone production. The patient consequently required levothyroxine replacement therapy.

The management of ectopic thyroid tissue depends on symptoms, thyroid function, the presence of other functioning thyroid tissue, and the potential risks associated with intervention. Asymptomatic patients with functioning ectopic thyroid tissue can generally be managed conservatively with clinical and biochemical monitoring. Levothyroxine replacement is indicated in patients with hypothyroidism and may also be considered in selected patients to suppress thyroid-stimulating hormone and limit enlargement of ectopic thyroid tissue [17]. Surgical excision is generally reserved for patients with significant obstructive or compressive symptoms, recurrent hemorrhage, suspicion of malignancy, or other specific indications.

Surgical management of a presumed TGDC in a child requires particular caution when the orthotopic thyroid gland cannot be demonstrated. The Sistrunk procedure remains the standard surgical treatment for TGDC [6]; however, if the lesion being excised contains the patient's principal functioning thyroid tissue, surgery can result in clinically significant hypothyroidism. This risk is particularly relevant in children because prolonged thyroid hormone deficiency can adversely affect growth and neurodevelopment [18].

An important feature of this case is that the preoperative CT had already raised the possibility of ectopic thyroid tissue and demonstrated the absence of the orthotopic thyroid gland. This emphasizes that identification of ectopic thyroid tissue on imaging should prompt functional assessment before proceeding with surgery. Although scintigraphy was ultimately performed postoperatively in this patient, preoperative functional assessment might have provided additional information regarding the distribution and functional status of thyroid tissue and potentially altered the management strategy.

A multidisciplinary approach involving otorhinolaryngologists, radiologists, endocrinologists, nuclear medicine specialists, and pathologists may facilitate accurate diagnosis and appropriate management. In children presenting with a midline neck swelling suggestive of a TGDC, careful identification of the normally located thyroid gland should form an important part of preoperative evaluation. If the orthotopic thyroid gland is absent or ectopic thyroid tissue is suspected, radionuclide scintigraphy should be considered before surgical excision whenever clinically feasible.

The present case highlights that a midline neck swelling with classical clinical features of a TGDC may contain ectopic thyroid tissue of substantial functional importance. Careful integration of clinical findings with anatomical imaging, thyroid function testing, cytology, and functional imaging is therefore essential to minimize the risk of inadvertent removal of functioning thyroid tissue.

## Conclusions

Ectopic thyroid tissue should be considered in the differential diagnosis of a midline neck swelling clinically suggestive of a thyroglossal duct cyst, particularly in children. The absence of the normally located thyroid gland on imaging should prompt further evaluation before surgical intervention. Although ultrasonography, CT, and FNAC can provide valuable anatomical and cytological information, assessment of thyroid function and, when indicated, radionuclide scintigraphy is important for determining the functional significance of ectopic thyroid tissue. In the present case, inadvertent excision of ectopic thyroid tissue during Sistrunk's procedure was followed by marked hypothyroidism requiring levothyroxine replacement. Careful preoperative identification and functional assessment of thyroid tissue may prevent this potentially avoidable complication.
